# Orthoplastic Approach to the Treatment and Reconstruction of a Neurotrophic Receptor Tyrosine Kinase Type 3 Soft Tissue Sarcoma Arising From the Occipitalis Muscle

**DOI:** 10.7759/cureus.74241

**Published:** 2024-11-22

**Authors:** Jay G Fiechter, Christopher Johnson, Justin Bryant, Joseph McCollom, Richard Zhang

**Affiliations:** 1 Orthopedics, Indiana University School of Medicine, Fort Wayne, USA; 2 Orthopedic Oncology, Orthopaedics Northeast, Fort Wayne, USA; 3 Craniofacial Reconstruction, Parkview Health, Fort Wayne, USA; 4 Medical Oncology, Parkview Cancer Institute, Fort Wayne, USA; 5 Medical Oncology, Parkview Health, Fort Wayne, USA

**Keywords:** hair-bearing tissue expansion, hair-bearing tissue flap, ntrk3 mutation, occipitalis muscle, orthoplastic surgery, soft tissue sarcoma

## Abstract

We present a multidisciplinary approach to the treatment of a neurotrophic receptor tyrosine kinase type 3 (NTRK3) soft tissue sarcoma (STS), arising from the occipitalis muscle. NTRK3 is a mutation only recently described in STS using next-generation sequencing and is rarely implicated in STS.Currently, there is limited literature to guide care. This case demonstrates a successful treatment option utilizing a multidisciplinary team and unique reconstruction with a hair-bearing scalp. To the best of our knowledge, this is the first case report showing an NTRK3 mutation STS arising from the occipitalis muscle. The utilization of staged hair-bearing tissue expansion post-wide R0 resection to reconstruct the scalp defect is a challenging reconstruction method.

We present a single clinical experience discussing a 40-year-old female with an NTRK3 mutation STS arising from the occipitalis muscle and involving the scalp. The diagnosis was made after the analysis of a punch biopsy specimen by a bone and soft tissue pathologist as a low-grade sarcoma harboring a sperm antigen with calponin homology and coiled-coil domains 1-like (SPECC1L)-NTRK3 fusion transcript. The patient underwent R0 resection by orthopedic oncology surgery and craniofacial microvascular plastic surgery. Staged reconstruction via hair-bearing tissue expansion was performed by the latter.

Eighteen months after the index procedure, no recurrent disease was detected, and the hair-bearing reconstruction was fully healed with well-concealed scars.

This case is a successful treatment method for a low-grade STS harboring a SPECC1L-NTRK3 fusion transcript. There is little published literature to guide care for low-grade NTRK3 mutation STS. This case highlights the importance of multidisciplinary care for STS.

## Introduction

We present a novel clinical experience discussing the surgical treatment and reconstruction of a neurotrophic receptor tyrosine kinase type 3 (NTRK3) soft tissue sarcoma (STS) arising from the occipitalis muscle. STSs harboring NTRK3 mutations are a rare and recently described new subtype. In addition, head and neck STSs are very rare, and this subtype has not been described as arising from the occipitalis muscle. Our clinical experience highlights the importance of multidisciplinary care and orthoplastic approach in providing successful treatment and reconstructive options for this challenging clinical scenario.

This article was previously presented as a poster at the 2023 Musculoskeletal Tumor Society Annual Meeting on October 4-6. 

## Case presentation

**Figure 1:**
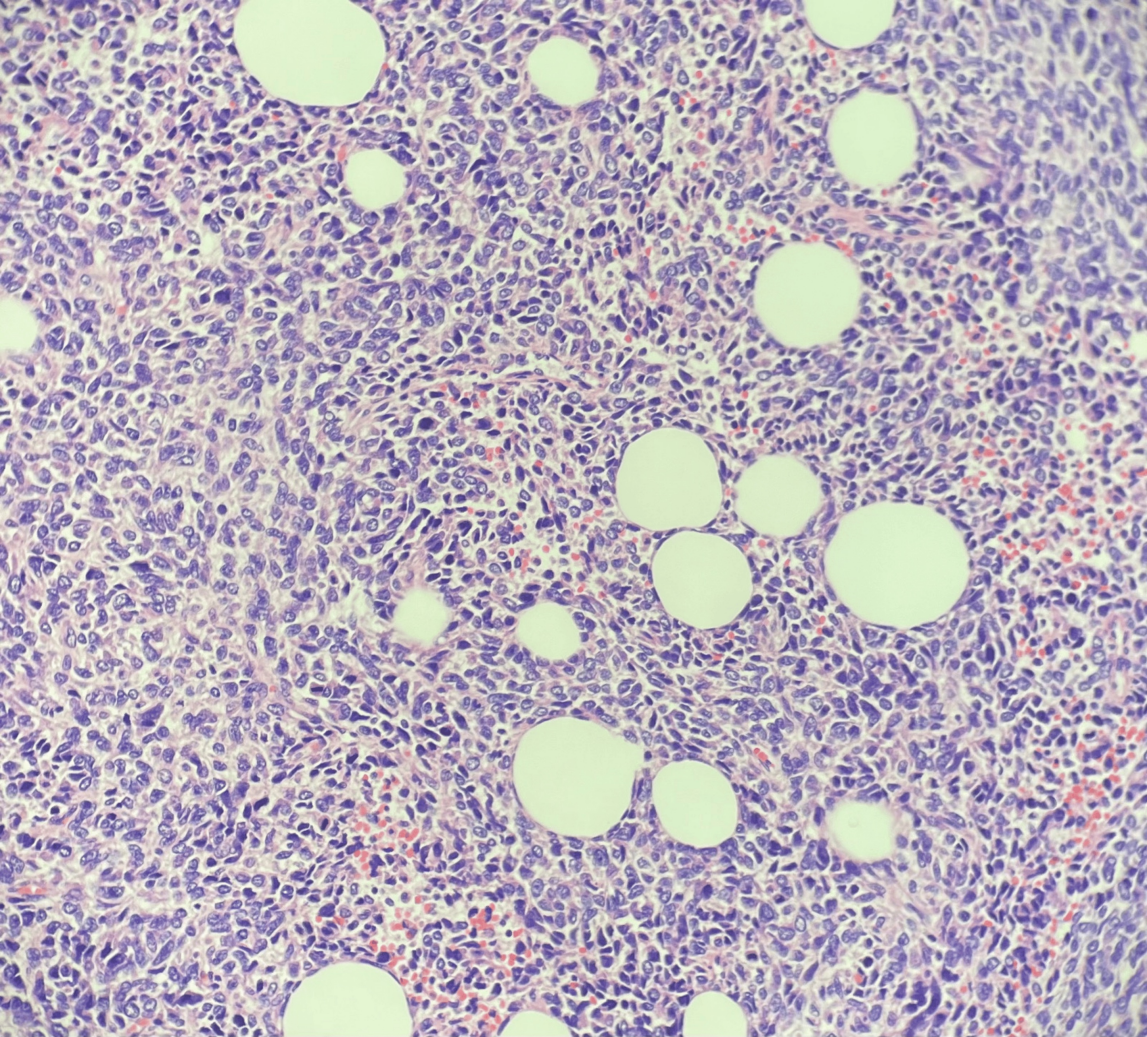
Histology of soft tissue sarcoma at 20× magnification demonstrating monotonous round to slightly elongated cells infiltrating the adipose tissue

A 40-year-old female presented to the sarcoma clinic with a tender, enlarging soft tissue mass arising from the left posterior scalp. Three months after initially noticing this nodular scalp mass, she presented to her dermatologist, where a punch biopsy was performed. Immunohistochemistry found the neoplastic cells to be variably positive for S-100 protein and strongly TRK positive. Desmin and CD31 were negative. The NTRK panel identified the sperm antigen with calponin homology and coiled-coil domains 1-like (SPECC1L)-NTRK3 fusion. The diagnosis was made by a bone and soft tissue pathologist as a low-grade sarcoma harboring a SPECC1L-NTRK3 fusion transcript (Figure 1).

**Figure 2:**
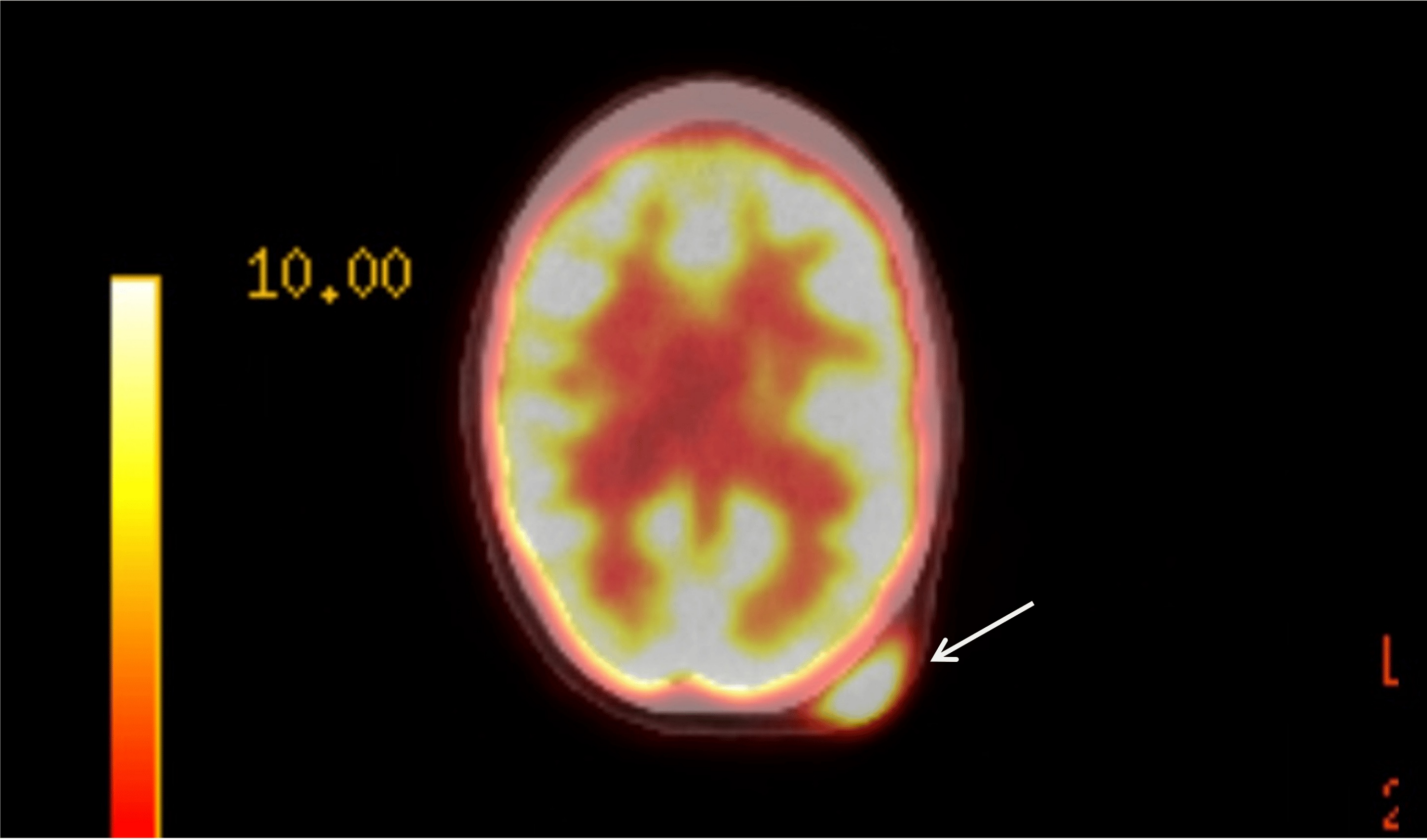
PET demonstrating FDG avid uptake in the arrow of known mass PET: positron emission tomography; FDG: fluorodeoxyglucose

**Figure 3:**
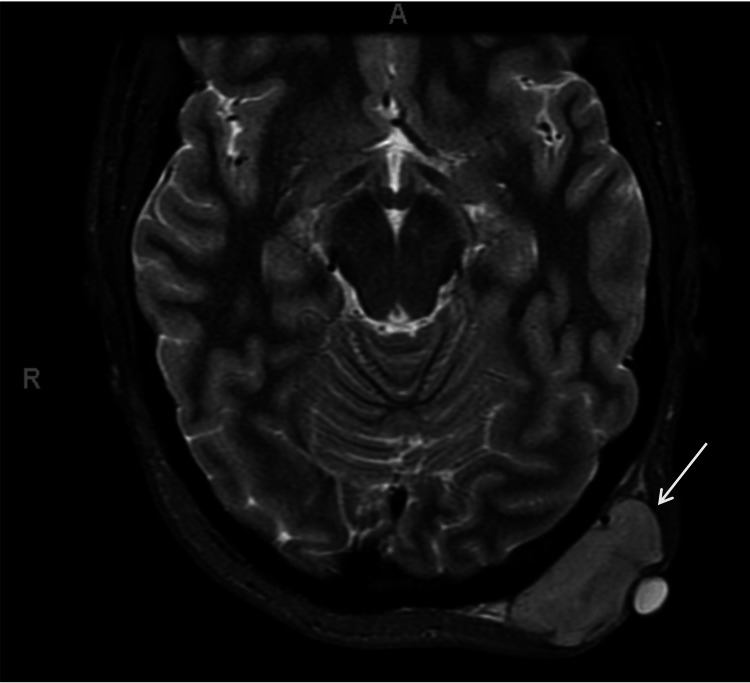
MRI demonstrating enhancing soft tissue mass without bony invasion (arrow) MRI: magnetic resonance imaging

A full body positron emission tomography (PET) scan and chest computed tomography (CT) were obtained and negative for metastatic disease. PET demonstrated a fluorodeoxyglucose (FDG) avid (standard uptake value (SUV) 13.1) soft tissue mass within the left occipitalis muscle (Figure 2). Magnetic resonance imaging (MRI) demonstrated an enhancing 5 cm heterogeneous soft tissue mass arising from the occipitalis muscle extending into the subcutaneous tissue without bony invasion (Figure 3).

The consensus recommendation at a multidisciplinary sarcoma tumor board was microscopically margin-negative (R0) resection. Radiation and systemic treatment were not recommended due to the low-grade STS. Based on the MRI, the patient would require subperiosteal resection of the cranium to ensure negative margins/fascial margins. Hair-bearing scalp reconstruction would be performed by craniofacial microvascular plastic surgery. 

**Figure 4:**
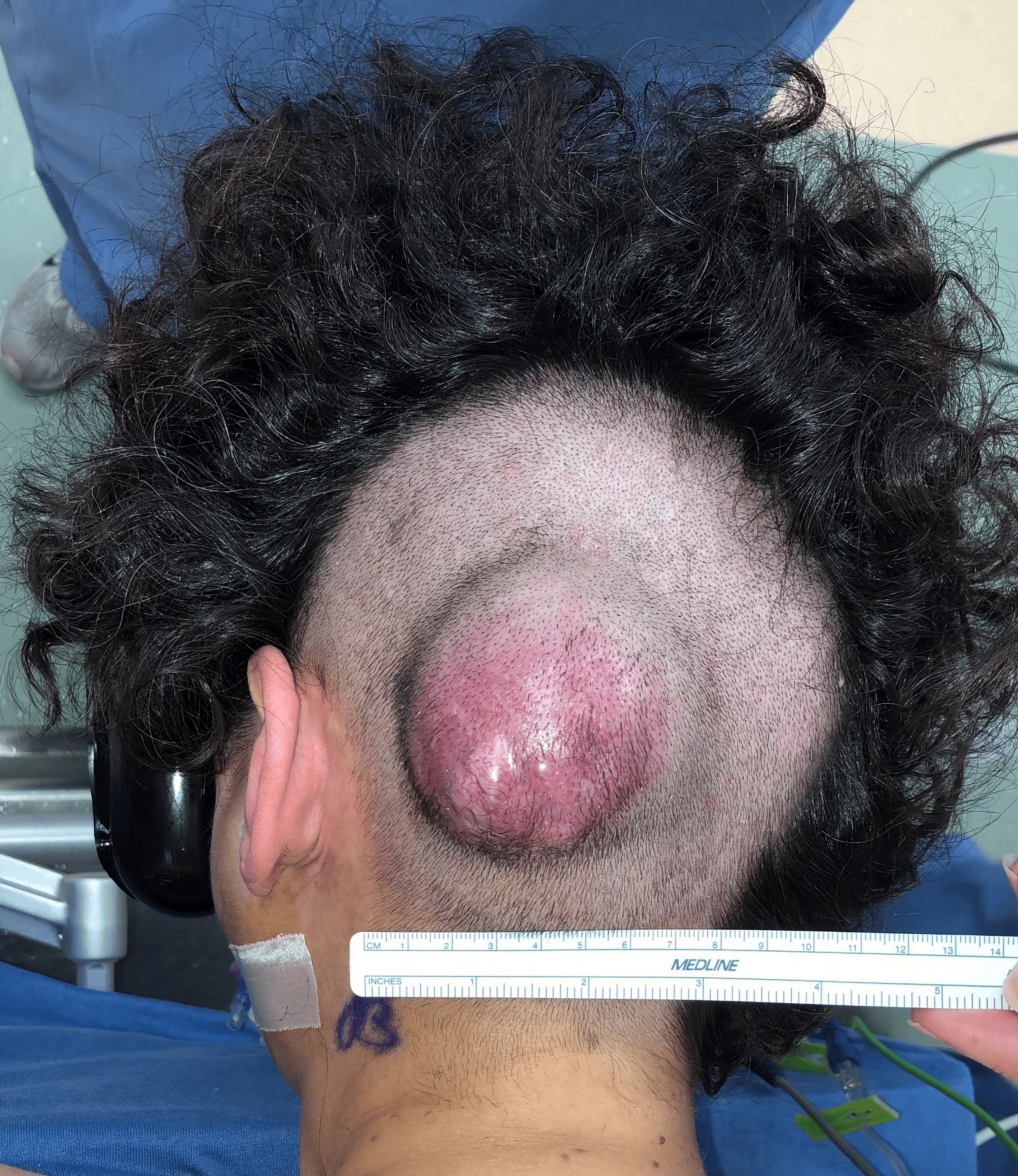
Gross occipital mass exposed after shaving the surrounding portions of the patient's head

**Figure 5:**
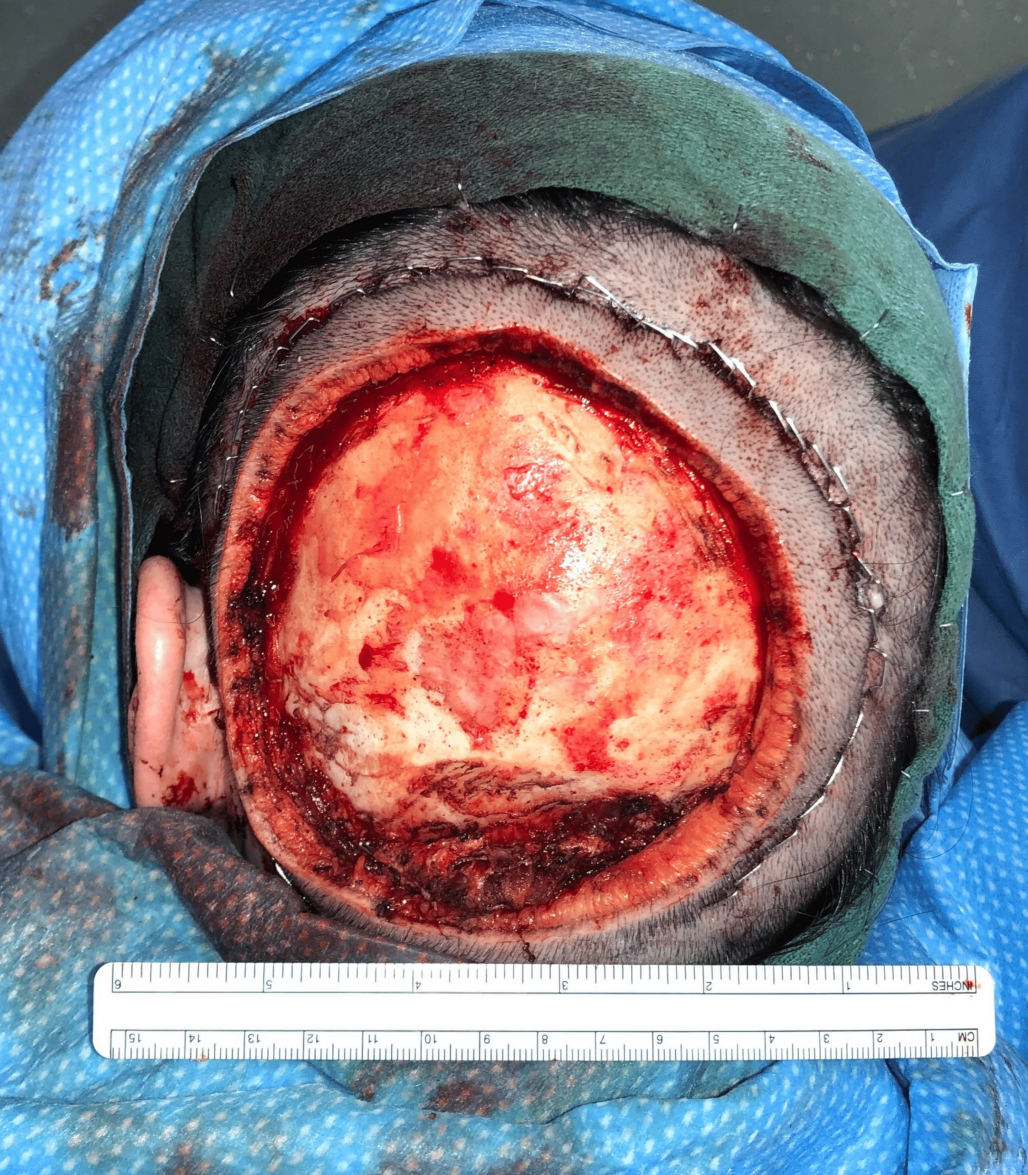
Wide surgical excision of soft tissue sarcoma with 2 cm margins including the pericranium at the deep margin

The surgical area was prepared by first shaving the patient's head, exposing the gross mass (Figure 4). A wide surgical excision of the left parietooccipital scalp STS with 2 cm margins, including the occipitalis muscle and pericranium at the deep margin, was performed. Strict hemostasis was established by circumscribing the soft tissue mass with staples per craniofacial microvascular plastic surgery, and orthopedic oncology removed the soft tissue mass (Figure 5). A synthetic acellular bilayer wound matrix was applied to the resultant 10.5×10 cm defect while awaiting the pathology evaluation of surgical margins. Histopathological examination confirmed a 6.3×5.6 cm low-grade sarcoma with SPECC1L-NTRK3 fusion and a mitotic rate of 20/10 per high-power field (HPF). All surgical margins were found to be free of tumor and without pericranial invasion. Hair-bearing scalp reconstruction via tissue expansion was pursued.

**Figure 6:**
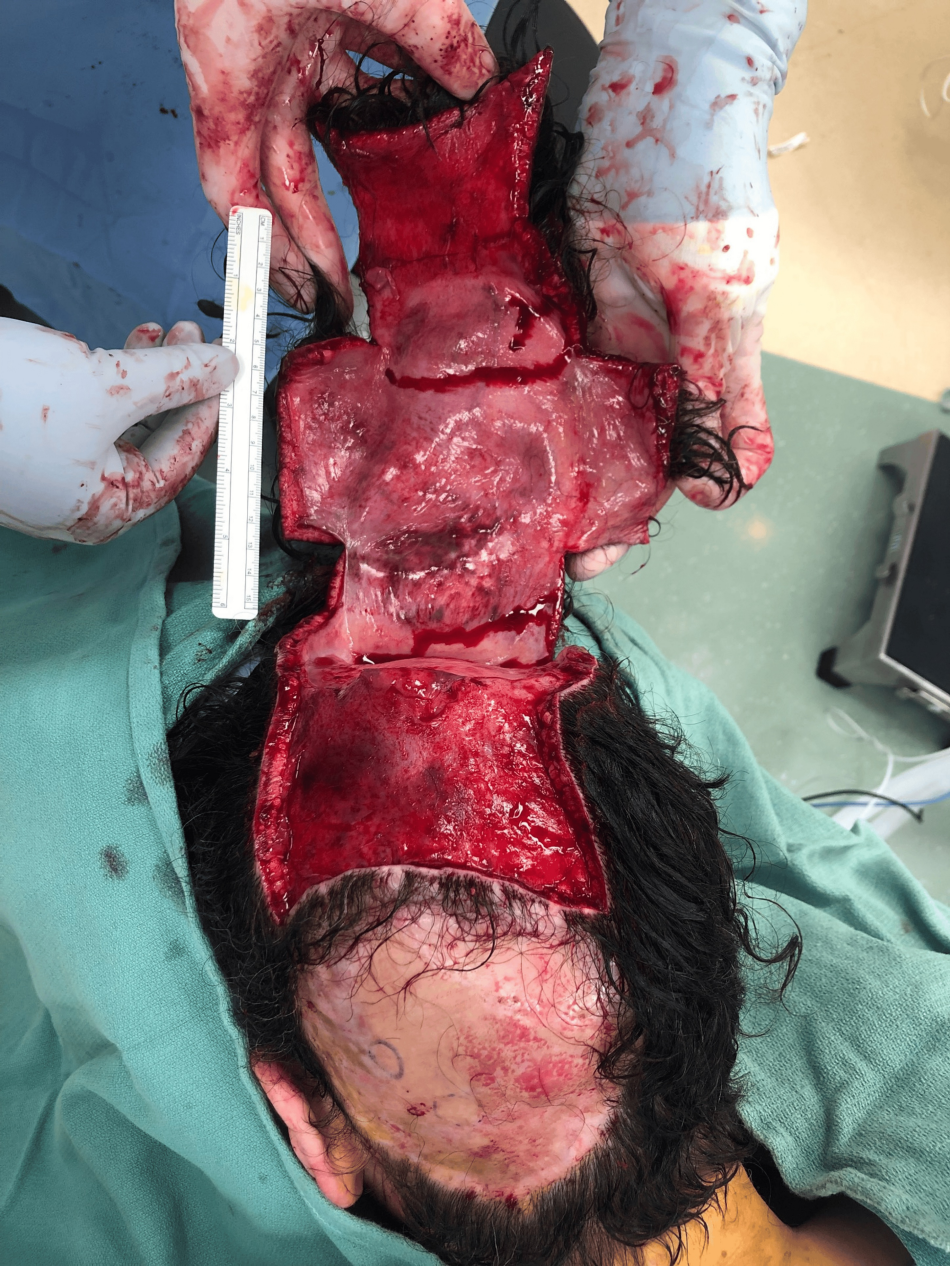
Flap reconstruction after successful tissue expansion

The patient progressed well with bilayer wound matrix incorporation, and three weeks after excision, an 11×11 cm split-thickness skin graft was applied to the surgical defect. A rectangular tissue expander was concurrently placed in the subgaleal plane of the parietal scalp. Tissue expansion was initiated three weeks postoperatively in-office and progressed to daily at-home tissue expansion. At a point midway through tissue expansion, the patient experienced exposure of the tissue expander. However, reconstruction was salvaged with the replacement of the tissue expander and coverage with the temporalis muscle flap beneath the scalp. Tissue expansion resumed three weeks later with at-home expansion to achieve the goal expansion volume of 600 cm^3^. After reaching the goal scalp expansion, the tissue expander was removed, the skin graft was excised, and the expanded parietal scalp was rearranged to reconstruct the parietooccipital defect, achieving complete reconstruction of the hair-bearing scalp (Figure 6).

**Figure 7:**
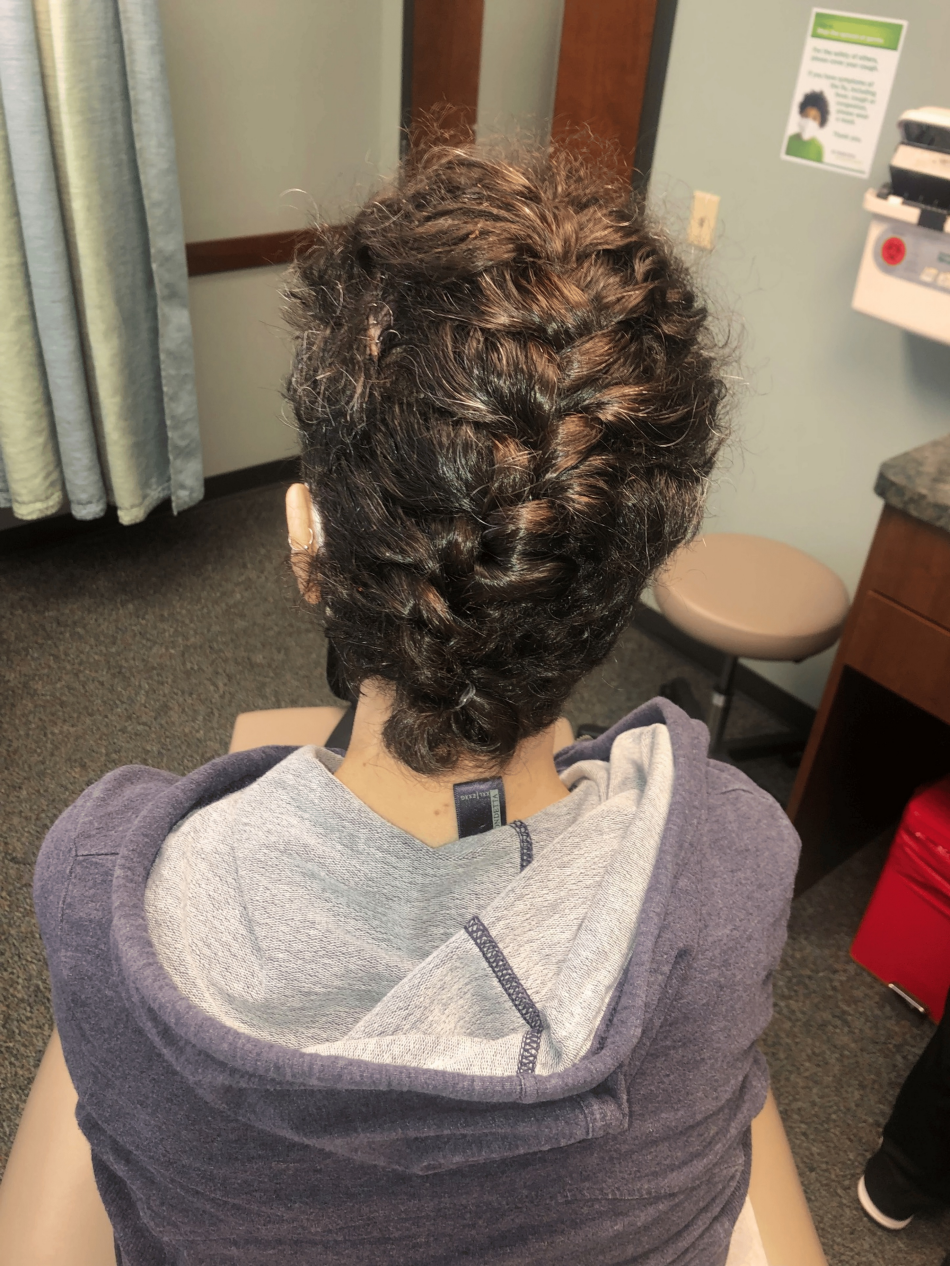
Image of the patient after the final hair-bearing flap reconstruction

Twenty months after the index procedure, no recurrent disease was detected, and the hair-bearing reconstruction was fully healed with well-concealed scars (Figure 7).

## Discussion

Currently, no randomized controlled trials dealing with the treatment of this specific STS subtype exist. The annual prevalence of STS is five per 100,000. The head and neck are the rarest anatomic locations [1-3], accounting for 5-15% of sarcomas and only 1% of malignant head and neck tumors in adults [4-6]. 

NTRK3 encodes a protein involved in the phosphorylation of the MAP kinase pathway [7]. NTRK3 is a mutation only recently described in STS using next-generation sequencing [8-11]. Mutations in this gene have now been described in 2.75% of all cancers. NTRK3 mutations have been implicated in only 2.01% of STSs [12]. 

Clinical evidence exists for the targeted treatment of higher-grade NTRK fusions using TRK inhibitors [13]. In a higher-grade fusion, one could consider TRK inhibitor use in a neoadjuvant setting to potentially reduce tumor size prior to surgery, minimizing the size of the defect and magnitude of reconstruction needed. Our patient's tumor was described as low-grade, and R0 resection without prior treatment was recommended.

The oncologic excision rendered the patient disfigured, requiring reconstruction. In considering reconstruction, tissue expansion has been utilized for a variety of reconstructive needs since the 1980s. An expander is placed beneath the subcutaneous or fascial plane, and expansion is performed in a stepwise manner by injecting liquid into the expander. The expander is then removed, and the expanded tissue components may be rearranged to reconstruct the defect [13]. This method has been used body-wide for multiple defects. Hair-bearing tissue expansion and reconstruction have been used in burn reconstructions, pediatric giant congenital nevus excision, as well as scalp defects after trauma [14,15]. Defects in hair-bearing areas often require much more precise tissue expansion and reconstruction. A 2020 case series discussed three cases where hair-bearing tissue expansion was utilized to reconstruct scalp defects [16]. Tissue expansion of the hair-bearing scalp in our clinical experience was able to achieve a nearly imperceptible scar resulting from an otherwise disfiguring oncologic surgical defect. 

## Conclusions

Our clinical experience demonstrates a successful treatment and reconstructive option for a low-grade STS arising from the occipitalis muscle. Hair-bearing tissue expansion allowed for an acceptable cosmetic result and oncologic outcome. This case expands the understanding of a relatively new STS subtype (NTRK3) arising from a newly reported anatomic location. Multidisciplinary care with orthoplastic approach optimized patient care and outcome in this challenging clinical scenario.
